# Human Papillomavirus Type 16 Entry: Retrograde Cell Surface Transport along Actin-Rich Protrusions

**DOI:** 10.1371/journal.ppat.1000148

**Published:** 2008-09-05

**Authors:** Mario Schelhaas, Helge Ewers, Minna-Liisa Rajamäki, Patricia M. Day, John T. Schiller, Ari Helenius

**Affiliations:** 1 Institute of Biochemistry, ETH Zurich, Zurich, Switzerland; 2 Department of Applied Biology, University of Helsinki, Helsinki, Finland; 3 Laboratory of Cellular Oncology, National Cancer Institute, National Institutes of Health, Bethesda, Maryland, United States of Maryland; Northwestern University, United States of America

## Abstract

The lateral mobility of individual, incoming human papillomavirus type 16 pseudoviruses (PsV) bound to live HeLa cells was studied by single particle tracking using fluorescence video microscopy. The trajectories were computationally analyzed in terms of diffusion rate and mode of motion as described by the moment scaling spectrum. Four distinct modes of mobility were seen: confined movement in small zones (30–60 nm in diameter), confined movement with a slow drift, fast random motion with transient confinement, and linear, directed movement for long distances. The directed movement was most prominent on actin-rich cell protrusions such as filopodia or retraction fibres, where the rate was similar to that measured for actin retrograde flow. It was, moreover, sensitive to perturbants of actin retrograde flow such as cytochalasin D, jasplakinolide, and blebbistatin. We found that transport along actin protrusions significantly enhanced HPV-16 infection in sparse tissue culture, cells suggesting a role for in vivo infection of basal keratinocytes during wound healing.

## Author Summary

To replicate, viruses have to enter into host cells. Since they have no means of locomotion, they rely entirely on cellular transport systems to access the cellular compartments where replication occurs. Following individual virus particles by video microscopy, we found that human papillomavirus type 16, the main causative agent of cervical cancer, bound to long finger-like protrusions of cells. There, they were transported from the periphery to the cell body. The transport was mediated by a process termed actin retrograde flow, where viruses bound to cell surface molecules hooked up to filamentuos actin and were dragged along with the actin-like items on a transport belt. Entry into the cell occured at the cell body. The results raised the interesting possibility that viruses use retrograde flow when they infect wounded epidermal keratinocytes, where finger-like protrusions of cells are abundant.

## Introduction

Virus entry into target cells is a multistep process that starts with the initial binding of incoming particles to cellular receptors and attachment factors. After binding, animal viruses typically display a period of lateral movement before viruses are internalized by endocytosis or penetrate the plasma membrane [1]. The dynamics of virus movement on the cell surface can be random as described for murine polyomavirus [2], or highly directional as described for murine leukemia virus and other enveloped RNA viruses [3]. In the latter case, movement was observed along actin-rich protrusions such as filopodia and retraction fibres, and the movement was dependent on actin retrograde flow that directed the virus toward the cell body. Here, we have analyzed the surface dynamics of Human Papillomavirus Type 16 (HPV-16) on tissue culture cells.

HPV-16 is a small non-enveloped DNA virus with an icosahedral (T = 7) capsid of 55 nm in diameter. HPV capsids contain two structural proteins, the major protein L1 that comprise the 72 pentamers, and the minor protein L2 that is principally located internally within the virion [4]. HPV-16 infection is linked to the development of cervical cancer. Infectious entry appears to occur specifically in the basal keratinocytes of mucosal epithelium subsequent to binding of virions to the basement membrane of a disrupted epithelium [5]. Since HPV replication and assembly requires infected basal keratinocytes to undergo the stepwise differentiation program of the epithelium [6], HPV propagation in cell culture is a major challenge. Surrogate production systems that generate infectious L1/L2 capsids containing marker plasmids, termed HPV pseudovirions (PsV), have been developed and successfully used to study aspects of HPV attachment and entry [7]–[13]. In this study, the HPV-16 PsV contained plasmids that upon successful entry expressed GFP or RFP.

Attachment and infectious uptake of several different HPV types requires heparan sulfate proteoglycans (HSPGs) [14]–[16]. However, a specific HSPG protein core does not seem to be required for HPV infection [17]. Recently, it was shown that HPVs also interact with extracellular matrix components such as laminin-5 or HSPGs [10],[11],[18],[19]. Productive entry involves internalization by endocytosis [20], a process that for HPV occurs slowly and asynchronously over a period of several hours [9],[21].

Prior to internalization, certain neutralizing antibodies no longer recognize the surface or lead to a release of bound viruses suggesting conformational changes in the capsid upon binding [10],[11],[13],[15],[21]. In addition, treatment of cell bound virus with DSTP27, a heparan sulfate binding drug, results in non-infectious internalization [11],[15],[21]. Hence, transfer to a secondary receptor has been proposed.

However, the dynamics of HPV interaction with the cell surface during the initial stages of infection are not understood. Using fluorescently labeled HPV-16 PsV that retained their infectivity, we therefore investigated the lateral mobility of capsids on the cell membrane by live cell imaging. Interestingly, several distinct modes of motion were observed including an active tranport towards the cell center.

## Results

### Fluorescent labeling of HPV-16 PsV

**Figure 1:**
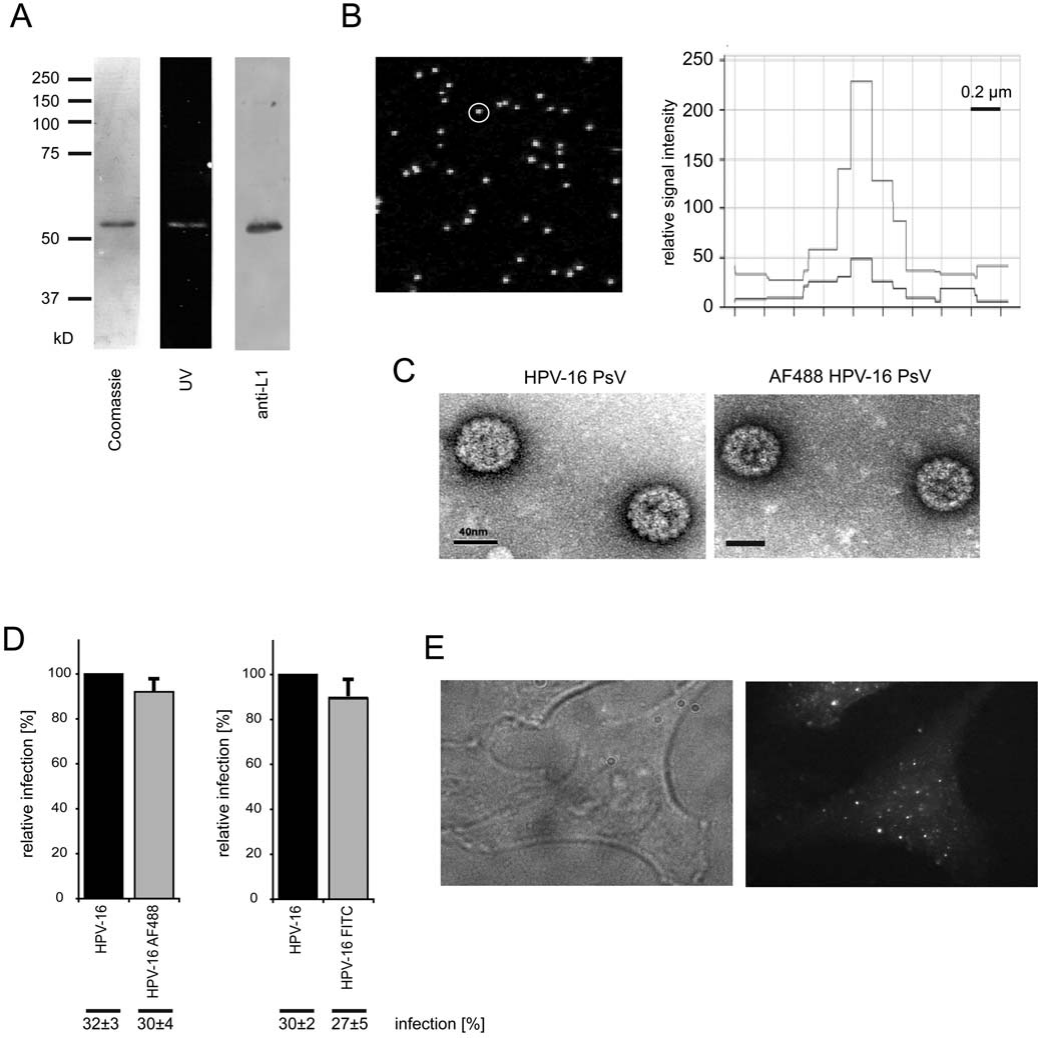
Covalent labeling of HPV-16 PsV with fluorophores. Purified HPV-16 PsV were covalently labeled with fluorophore-(AF488, FITC)-succinimidylester as described in Materials and Methods. After purification the labeled PsV were analyzed as follows. (A) SDS-gel electrophoresis of AF488 labeled HPV-16 PsV, from left to right: coomassie staining, UV emission, western blot with an antibody directed against L1. (B) AF488 labeled HPV-16 PsV particles bound to glass coverslips, confocal microscopy image (left). Depicted is a representative fluorescence intensity profile (right) of the white encircled AF488 labeled HPV-16 particle (left). The fluorescent signal of AF488 HPV-16 particles matched the signals of labeled Simian Virus 40 particles that are similar in size indicating the absence of bigger aggregates (not shown). (C) Electron micrographs of negatively stained HPV-16 PsV (top), or AF488 labeled HPV-16 PsV (bottom). Bars represent 40 nm. (D) HeLa cells were inoculated with either unlabeled or fluorophore labeled HPV-16 PsV (each about 50 particles/cell) to result in about 30% RFP expressing cells. The percentage of cells expressing RFP (infected cells) was determined by flow cytometric analysis 24 h post inoculation after trypsinization and subsequent fixation by 4% formaldehyde. The graph shows the relative amount of infected cells normalized to the unlabeled virus preparation. Error bars represent the standard deviation of three independent experiments. Numbers below the graph indicate unnormalized infection data (infection). (E) Wide field microscopy of a HeLa cells inoculated for 5 min (37°C) with AF488 labeled HPV-16 PsV (100 particles/cell) with DIC image (left), and AF488 fluorecence (right).

To visualize the behaviour of cell-bound viruses, purified HPV-16 PsV were covalently labeled with the fluorophores AF488 or FITC. About 200 fluorophores per particle were covalently attached mainly to the major capsid protein L1 (Fig. 1A, see Materials and Methods). A homogeneously labeled particle suspension was obtained as indicated by confocal microscopy of labeled particles attached to glass coverslips (Fig. 1B, left). The fluorescent signal intensity profile of spots followed a single Gaussian distribution, which indicated that light is emitted from single particles (Fig. 1B, right). The signal had a diameter of 0.2–0.4 µm, similar to other viruses of a comparable size [22],[23]. The virion structure remained unchanged as judged by negative staining and electron microscopy (Fig. 1C). To test whether labeling would affect the entry properties, we compared labeled and unlabeled HPV-16 PsV in their ability to express RFP (infection) from the incorporated plasmid by flow cytometry. We found that the number of infected cells remained virtually unchanged indicating that the particles labeled with fluorophores were fully entry competent (Fig. 1D).

### Mobility of HPV-16 PsV on the cell surface

**Figure 2:**
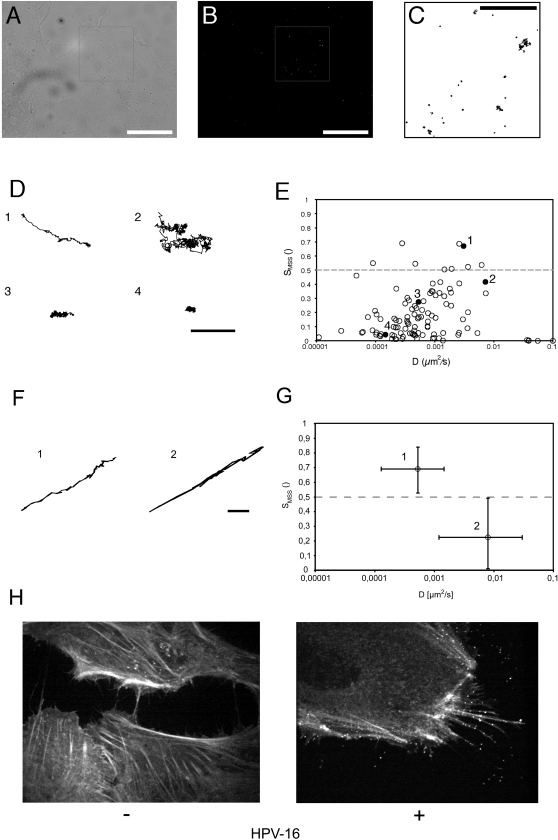
HPV-16 cell surface dynamics. HeLa cells were inoculated on 18 mm coverslips with HPV-16 PsV (about 100 particles/cell) for 5 min (A–E, H), or for 5–120 min (F, G) at 37°C prior to acquisition of images. (A) Differential interference contrast (DIC) picture of the cell shown in (B). (B) TIRF-M image from a time series (20 Hz, 1000 frames). (C) Trajectories of surface bound HPV-16 PsV from the inset (A, B) were detected by AF488 fluorescence (Scale bar for A–C 10 µm). (D) Representative trajectories of the four different modes of motion: (1) directed movement, (2) constraint diffusion, (3) slow drift, and (4) stationary particle (scale bar 1 µm). (E) Scatter plot of the diffusion coefficient versus the slope of the moment scaling spectrum (S_MSS_) of HPV-16 PsV trajectories. Every point represents one trajectory. The dots marked 1–4 represent the virus trajectories shown in (D). (F) Representative trajectories of HPV-16 PsV on cellular extensions in two different modes of motion: (1) directed movement, (2) constraint diffusion (Scale bar 1 µm). (G) Scatter plot of the diffusion coefficient versus the slope of the moment scaling spectrum (S_MSS_) of HPV-16 PsV trajectories on cellular extensions. Points represent the median of all trajectories that have a value for the S_MSS_ either above or below 0,5. Error bars represent the lowest or highest respective values of the two populations (S_MSS_>0.5, n = 29; S_MSS_<0.5, n = 27). (H) Confocal images of HeLa cells expressing EGFP-actin, with (right, 50 particles/cell) or without (left) HPV-16 PsV bound.

When labeled PsVs were added to cells at 37°C, binding was readily observed by fluorescence microscopy (Fig. 1E). The majority of virus particles that bound to cells did so within five minutes (not shown). While most of the viruses bound to the top surface of cells, some drifted into the narrow space between the cell and the cover glass, where they bound to cells. These viruses could be visualized by total internal reflection fluorescence microscopy (TIRF-M), and their movement could be followed by video microscopy (Fig. 2A, B).

**Figure 3:**
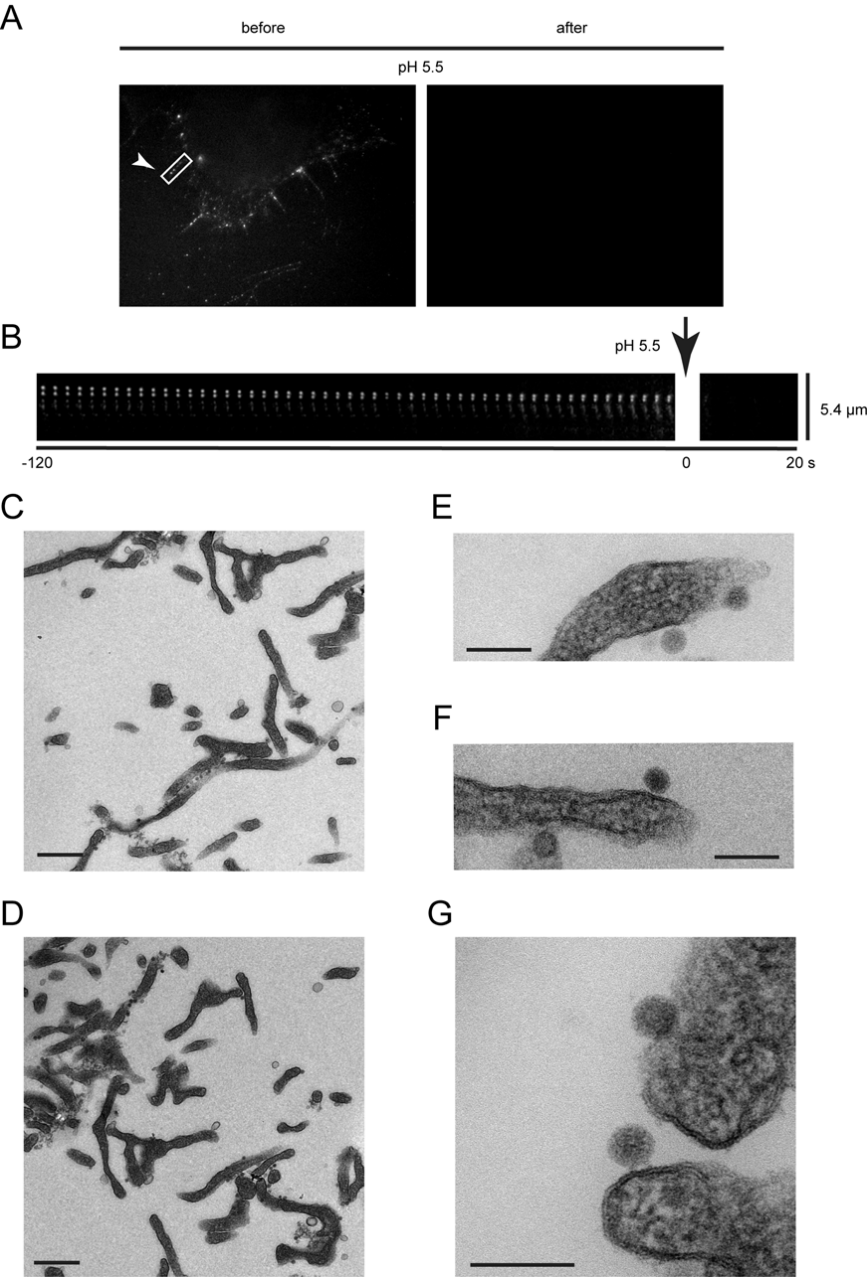
HPV-16 binds to and is transported on cell protrusions extracellularly. HPV-16 PsVs were covalently labeled with FITC and about 40 particles /cell were added to HeLa cells for 5 min. prior to image acquisition by epifluorescence microscopy at 2 fps. After 200 frames the extracellular medium was acidified to pH 5.5. (A) Stills show the FITC channel prior to and after acidification. (B) The boxed area of (A) was converted to a kymograph over 240 frames showing every fourth frame with the bottom representing the cell body. (C–G) HeLa cells were inoculated with HPV-16 PsV for 10 min (C, E, F) or 1 h (D, G) and processed for thin section electron microscopy. Viral particles were found to bind to cellular protrusions exclusively outside of the plasma membrane. Bars represent 500 nm (C, D) or 100 nm (E–G).

**Figure 4:**
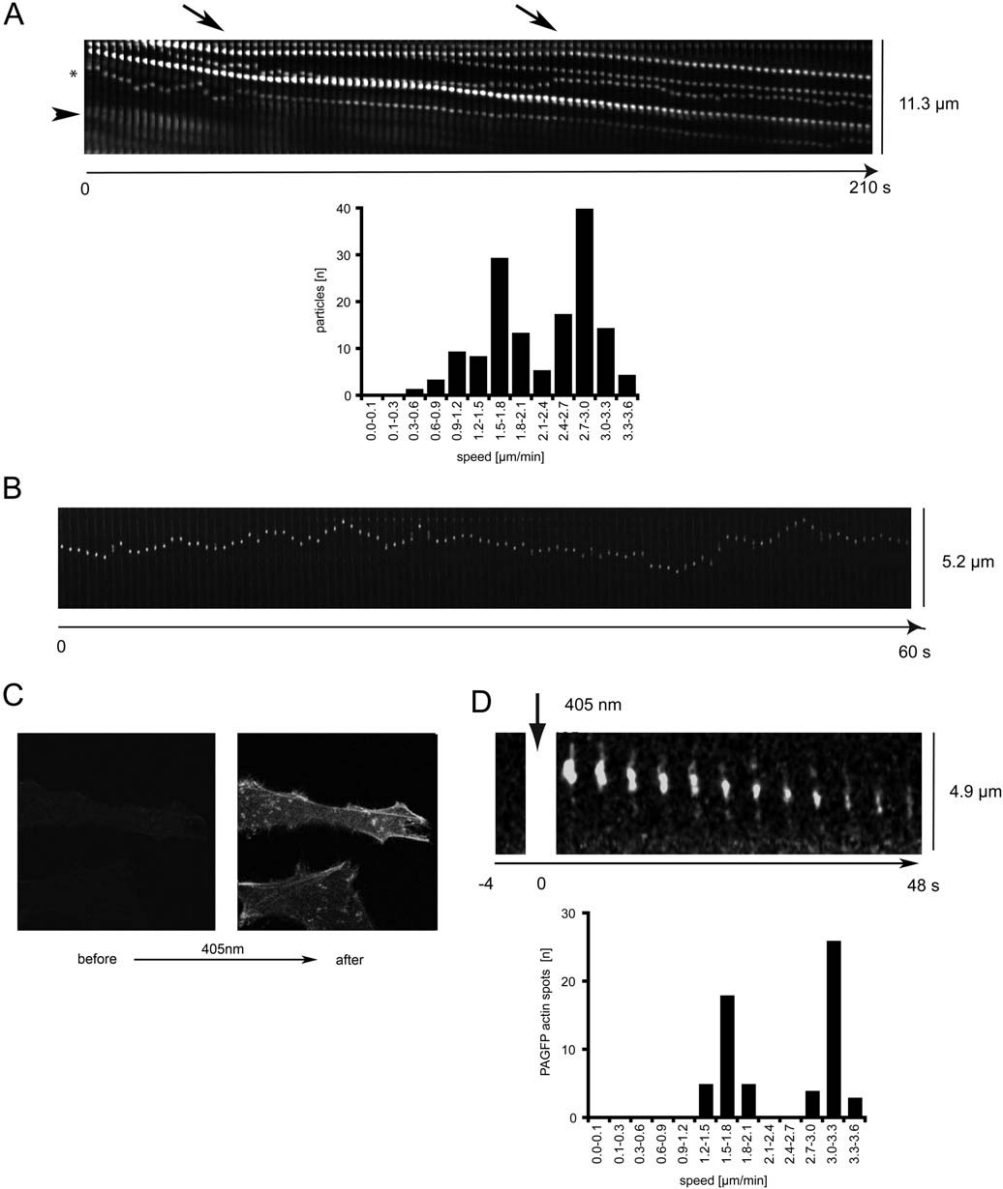
Directed virus movement along actin protrusions is slow and coincides with actin retrograde flow. HeLa cells were transfected with EGFP-actin and subsequently inoculated with AF488 labeled HPV-16 PsV (20–100 particles/cell) or SV40 (10 particles/cell). 20 min after virus addition cells were imaged at 2 fps with spinning disc confocal microscopy. (A) Kymograph shows pictures of a single actin protrusion of HeLa cells with several HPV-16 particles bound from time series (2 s intervals) oriented such that the tip of a protrusion is at the top and the cell body at the bottom. The slopes of viral particle location over time reflect the speed by which particles moved from the tip to the bottom of an actin protrusion. The speeds for 242 particles in directed motion from 35 movies were calculated, and displayed as the number of particles moving within a certain speed category. (B) Kymograph shows pictures from time series of a single actin protrusion of HeLa cells with a SV40 particle bound. (C) Confocal image of a single HeLa cell expressing beta-actin tagged with photoactivatable GFP prior to and after activation of PAGFP with short wavelength (405 nm) light. (D) Kymograph of a single protrusion of a HeLa cell expressing PAGFP-actin that was spot-activated with focussed short wavelength (405 nm) laser light. The GFP emission of the single spot showed an F-actin patch moving towards the cell body (bottom) reflecting retrograde flow. The speed of actin retrograde flow within protrusions was calculated and displayed as the number of spots moving within a certain speed category.

When HPV-16 particles were bound to the bottom surface of cells, and followed on the cell body by video TIRF-M at 20 frames per s for a total of 100 s (Videos S1, S2, S3, S4, S5; Fig. 2A–C), it was apparent that the movement of individual particles was heterogeneous. Trajectories extracted from digital image series using a single particle tracking (SPT) algorithm [24] showed that the majority of particles were essentially immobile, or displayed a slow drift (Fig. 2C, D; 3, 4). Some were, however, highly mobile displaying either random or relatively linear trajectories (Fig. 2C, D; 1, 2).

To describe the dynamic events and lateral mobility of bound HPV-16 PsV more quantitatively, we used a recently described algorithm that allowed definition of rate and mode of motion for each particle [2]. For convenient access of data in one graph we plotted the linear diffusion coefficients (D), a measure of the particle speed, and the slope of the moment scaling spectrum (S_MSS_), a measure for the mode of movement, for each viral particle. An S_MSS_ value of 0.5 defines random, Brownian movement, whereas values below and above 0.5 are characteristic of confined and directed movement, respectively, with an S_MSS_ value of 0 for immobility [2]. When we plotted the D vs. the S_MSS_ values for the recorded particles (n = 100), four modes of motion could be distinguished: (i) confinement (S_MSS_<0.1; Fig. 2E, 4), (ii) confinement with a slow drift (D<0.001 µm^2^/s, S_MSS_ = 0.15–0.35; Fig. 2E, 3), (iii) fast random motion with transient confinement (D>0.002 µm^2^/s, S_MSS_<0.5; Fig. 2E, 2), and (iv) ballistic, directed movement of PsV (S_MSS_>0.5; Fig. 2E, 1). Overall, the modes and speeds of motion of viral particles on the cell were comparable to those previously observed for murine polyomavirus [2], a small nonenveloped DNA virus that binds to the glycolipids GD1a and GT1b, [25],[26]. However, some HPV-16 particles exhibited, in addition, directed motion such as those shown in Fig. 2D, 1.

When analyzed in more detail, we found that directed motion of viruses observed on the cell body occured much more frequently on finger-like cell protrusions such as filopodia or retraction fibres. Since these protrusions were not always close enough to the cover glass to be easily visualized by TIRF-M, we used wide field or spinning disc confocal microscopy, which allowed us to image the protrusions over their full lengths with only a small decrease in acquisition speed. Protrusions of HeLa cells with HPV-16 PsV bound for 5–120 min were imaged at 2–5 frames per s, virus trajecories were extracted, and their mode of motion was analyzed quantitatively as described above. When we plotted the D vs. the S_MSS_ values for the recorded particles, predominantly two modes of motion along the cell protrusions could be observed regardless of the time after binding of the virus: (i) directed particle motion (S_MSS_>0.5, n = 29) (Fig. 2 F, G, 1), and (ii) random motion restrained by the width of protrusions to an almost 1-dimensional diffusion (D>0.002 µm^2^/s, S_MSS_<0.5, n = 27) (Fig. 2 F, G, 2), with the number of confined particles limited to a fraction below 10% (not shown).

### HPV-16 moves along actin protrusions on the outside of the plasma membrane

We surmised that the finger-like cell protrusions constituted filopodia or retraction fibres, because they contained actin in cells transiently transfected with GFP-actin (Fig. 2 H, Video S6). The binding to and movement along actin-rich protrusions of HPV-16 PsV was reminiscent of the retrograde movement described for several enveloped RNA viruses [3]. These RNA viruses bind to cell receptors and are transported along actin rich protrusions on the outside of the plasma membrane.

To determine whether HPV-16 was transported extracellularly along these protrusions, we tested whether the fluorescence of FITC labeled HPV-16 migration was diminished by acidification of the extracellular medium. FITC is a pH sensitive fluorophore that loses its fluorescent properties, when exposed to a pH below 6.0 due to protonation [27]. Since the fluorescence of particles was quenched upon acidification, we concluded that the HPV-16 PsV were moving along the outside of the actin protrusions (Fig. 3A, B, Video S7).

Thin section electron microscopy confirmed that the particles were located exclusively on the outside of the plasma membrane when associated with these narrow cell protrusions (Fig. 3, C–G). Unlike Simian Virus 40, a structurally similar polyomavirus that is observed juxtaposed to the plasma membrane [28], a gap of 12 nm±4 nm was seen between the surface of the particle and the plasma membrane arguing that they had bound to a membrane receptor with a large or extended ectodomain (Fig. 3, E–G). Occasionally, strands of electron dense material were observed inbetween virus and the plasma membrane most likely representing receptor molecules (Fig. 3G, arrowheads).

### HPV-16 transport kinetics coincide with actin retrograde flow

HPV-16 transport along actin protrusions on the outside of the plasma membrane is reminiscent of the transport of polyethylenimine-coated beads [29], of murine leukemia virus [3], and the epidermal growth factor (EGF) receptor [30],[31]. These transport phenomena have been attributed to actin retrograde flow, which causes the net transport of actin molecules within filaments from the plus end at the tip of an actin protrusion towards the minus end at the cell body. To find out whether HPV-16 transport may equally be powered by actin retrograde flow, we analyzed the speed of HPV-16 transport and compared it to the speed of actin retrograde flow.

To determine the speed of virus movement, we analyzed time lapse movies of individual viruses using kymographs. Individual actin protrusions were oriented such that they reflected a linear track with the tip at the top and the cell body at the bottom. Images were assembled consecutively. The mode of motion could easily be distinguished in the kymographs: directed transport towards the cell body is marked by virus particles on a straight line with a negative slope, particles diffusing randomly are visible as ragged, horizontal line, and confined particles appear aligned on a straight horizontal line (Figure 4A). Interestingly, when fluorescently labeled vesicular stomatitis virus (VSV, not shown), Semliki Forest Virus (SFV, not shown), or Simian Virus 40 (SV40, Fig. 4B, Video S8) were added to cells, only diffusive motion was observed suggesting that the directed motion of HPV-16 was a specific receptor-mediated process. The slope of HPV-16 particles in kymographs represented the speed of particle movement. Figure 4A shows the movement of several virus particles along a single actin protrusion. The speed of virus particles was slow and averaged 2.2±0.8 µm/min (n = 242). The speeds for actin retrograde flow and RNA viruses moving along actin protrusions are 1–5 and 2 µm/min, respectively [3],[29],[32], in line with our assumption that HPV-16 PsV were transported by actin retrograde flow.

Short stationary periods were also observed for single particles while other particles continued to move (Figure 4A, arrows). These stationary periods may have resulted from obstacles or interactions of virus with extracellular matrix components. However, when virus particles continued to move, they exhibited exactly the same speed as before.

Since the speed of virus particles in directed motion was always identical on the same actin protrusion (Figure 4A), we plotted the speed versus particle number, and found a bipolar speed distribution with two maxima at 1.6 and 2.9 µm/min. The result suggested that two kinds of actin protrusions in HeLa cells existed, and that viruses moved on one kind at about half of the speed than on the other.

Occasionally, we observed HPV-16 particles moving in a diffusive mode of motion, which subsequently switched to directed motion (Fig. 4A, asterisk). The opposite was never observed under unperturbed conditions. However, the switch from diffusive to directed motion was observed at different times post addition of virus, and several particles exhibited diffusive motion as long as 2 h post addition of virus. This indicated that HPV-16 particles required a trigger for directed motion, but possibly not all particles were able to switch from diffusive to directed motion.

To compare virus transport with the speed of actin retrograde flow we made use of a photoactivatable GFP-actin (PAGFP-actin) fusion construct. Activation of PAGFP was achieved by a brief pulse of short wavelength light, after which the illuminated PAGFP exhibited the fluorescent properties of normal GFP [33]. Expression of PAGFP-actin resulted in incorporation of the molecules into actin filaments (Fig. 4C). When we now activated a spot of PAGFP-actin in actin protrusions and followed the GFP spot over time, we found that this spot moved towards the cell body (Fig. 4D). The fluorescent intensity of the spot decreased over time, which was probably due to photobleaching and diffusion of G-actin. However, we consistently observed a retrograde movement of the spot, that represented activated PAGFP-actin molecules present in the actin filaments, and that could be used to analyze the speed of actin retrograde flow (Fig. 4D, Video S9). When we analyzed the speed of actin retrograde flow as before, we found a bipolar speed distribution of actin retrograde flow with maxima at 1.7 and 3.2 µm/min (average 2.5±0.9 µm/min, n = 63) that matched the speed of virus particle movement (Fig. 4A, D).

That virus transport and actin retrograde flow occured at the same rate was also suggested by the movement of EGFP-actin speckles infrequently observed in our virus transport kymographs. These speckles resulted from a patchy incorporation of EGFP-actin molecules into actin filaments [34], which, in turn, gave rise to an increased EGFP-actin fluorescence in certain regions of cell protrusions (Fig. 4A, arrowhead). Taken together, these results support the concept that virus transport was connected to the net transport of F-actin.

### Decrease of actin retrograde flow results in abrogation of virus transport

Actin retrograde flow is the result of basically three processes. First, actin polymerisation occurs at the tip of filaments and this pushes the filaments towards the cell body. Second, anchored myosin II pulls actin filaments towards the cell body. And third, depolymerisation and fragmentation of actin filaments reduces the barrier tension of the actin cortex-filament interface at the cell body and thus facilitates filament transport towards the cell body. When the three processes are in balance, the length of the actin filaments remains constant with a net transport of individual actin molecules present in the filaments towards the cell body [29],[35].

**Figure 5:**
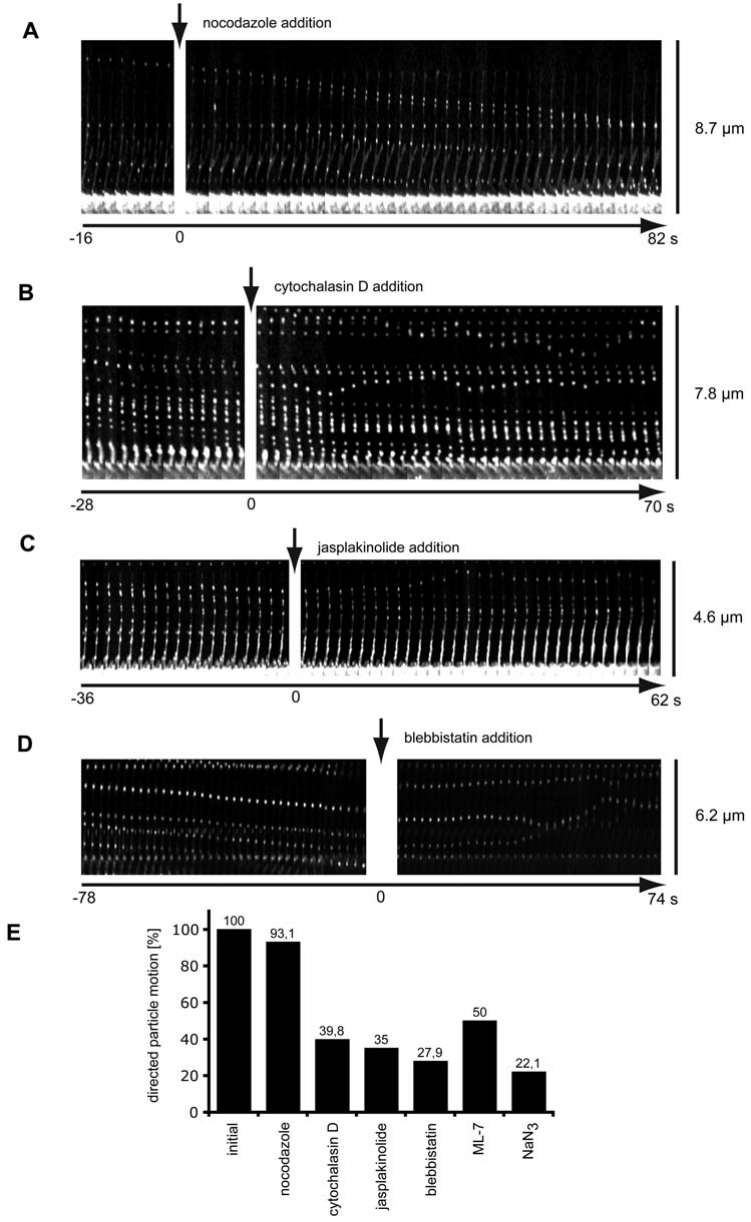
Pertubation of actin polymerisation dynamics and myosin II function abrogates virus transport. HeLa cells were transfected with EGFP-actin and subsequently inoculated with HPV-16 PsV (20–100 particles/cell). 5–15 min after virus addition cells were imaged at 2 fps with spinning disc confocal microscopy. About 1 min after image acquisition started various inhibitors were added as indicated. Kymographs show pictures of single actin protrusions of HeLa cells from time series (2 s intervals) oriented such that tip of protrusions are at the top and the cell body at the bottom. (A) Addition of the microtubule dissociating agent nocodazole (5 µM) does not perturb directed virus movement or protrusion structure. (B) Addition of the actin depolymerising agent cytochalasin D (2 µM) slows directed virus movement over a time period of 10–60 s whereafter the virus is either stuck or switches to random diffusion. (C) Addition of the actin stabilizing drug jasplakinolide (300 nM) similarly slows directed virus movement over a time period of 10–60 s whereafter the virus is either stuck or switches to random diffusion. It is interesting to note that the signal for actin frequently increased in the lower half of the protrusion. (D) Addition of the specific myosin II inhibitor blebbistatin (30 µM) almost instantaneously abrogated directed virus movement whereafter the virus more frequently diffused randomly on the protrusion. (E) Summary of the effects of inhibitors: The number of virus particles initially detected in directed motion was set to 100% and compared to the number of particles that still moved in a directed fashion after 80 s (inhibitor concentrations: 50 µM for ML-7, 10 mM for sodium azide). The number of particles analyzed was as follows: nocodazole, n = 143; cytochalasin D, n = 262; jasplakinolide, n = 33; blebbistatin, n = 219; ML-7, n = 39; sodium azide, n = 27.

To functionally adress the role of actin retrograde flow in virus transport along actin protrusions, we analyzed the contribution of ATP production, actin polymerisation, depolymerisation, myosin II function, and, as a control, microtubule stability using pharmacological inhibitors. We found that microtubule dissociation by nocodazole had no effect on virus transport (Fig. 5A, Video S10). However, cytochalasin D, which inhibits actin polymerisation by binding to the barbed ends of F-actin, and leads to actin depolymerisation by fragmentation of F-actin upon longer exposure, decelerated virus transport over a time period of 5–40 sec. Afterwards the virus particles frequently exhibited stationary behaviour or diffusive movement along the actin protrusions (Fig. 5B, Videos S11, S12). The same was observed when jasplakinolide (Fig. 5C, Video S13), a F-actin stabilizer and inducer of actin polymerisation, or sodium azide (ATP depletion, not shown) were added. The strongest effect was observed when myosin II function was inhibited by either blebbistatin (Fig. 5D, Videos S14, S15) or the myosin light chain kinase inhibitor ML-7 (not shown); virus transport stopped almost instantaneously and the frequency of particles that switched to a random diffusive mobility was the highest. Seldomly, particles were observed to move in an outwards direction, which was probably due to filopodial outgrowth induced by inhibition of myosin II [36],[37].

When we analyzed how many of the directionally mobile particles switched to a confined or diffusive mode of motion, we found that 50% or more virus particles lost their directed motion pattern within 80 s after treatment with inhibitors of actin retrograde flow, as compared to 7% in the nocodazole treated control samples (Fig. 5E). This indicated that virus transport was, indeed, functionally linked to actin retrograde flow. It was interesting to note, that on a single filopodium virus transport was either abrogated for all particles or all particles continued to move with decelerated speeds. With respect to inhibitor sensitivity, there was no significant difference for particles that moved at 1.6 µm/min or 2.9 µm/min suggesting that both forms of actin retrograde flow, the slower and the faster, are mechanistically similar.

### Virus tranport along filopodia contributes to infection

To determine whether the movement of virus particles along actin protrusions was involved in productive infection, we tested the effect of the myosin II inhibitor blebbistatin on infection.

**Figure 6:**
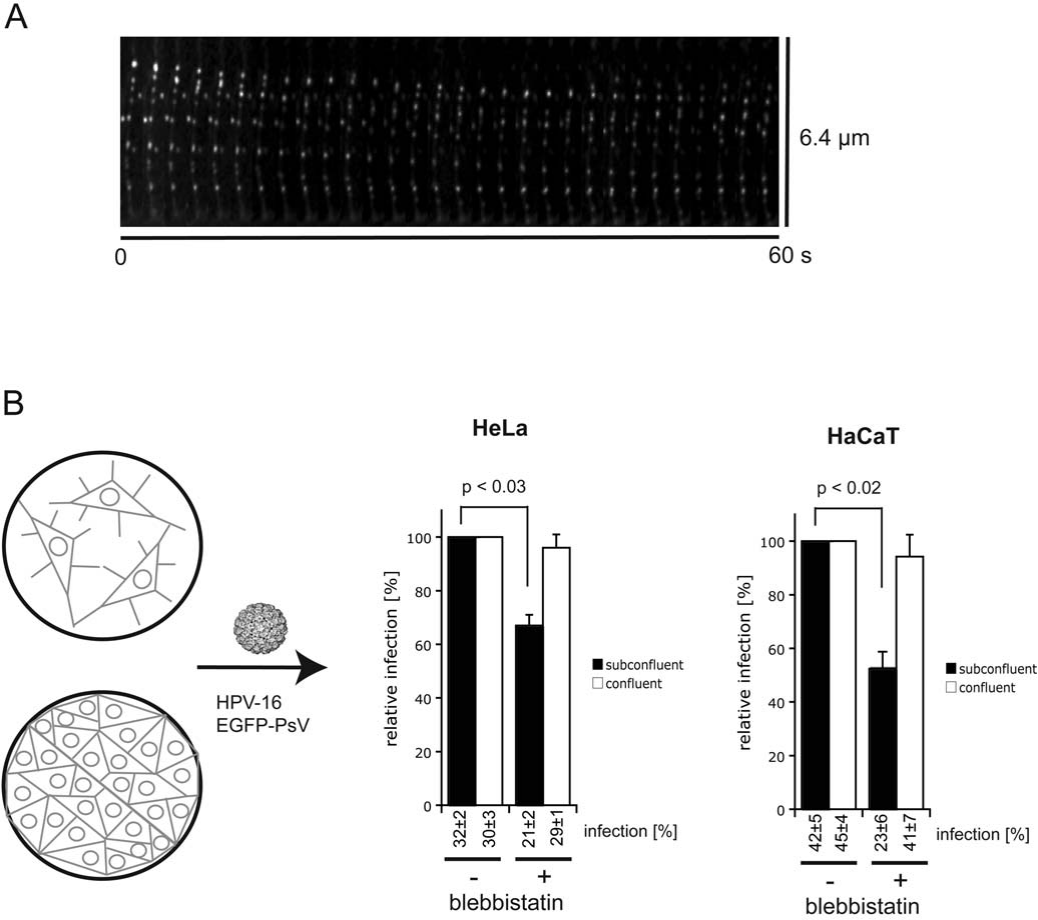
Inhibition of virus transport in keratinocytes and HeLa cells reduces the efficiency of infection. (A) HeLa cells were infected with HPV-16 PsV (about 50 particles/cell) expressing GFP with or without inhibition of actin retrograde flow by blebbistatin. Cells were grown as confluent monolayers without the presence of long actin protrusions or subconfluently exhibiting frequently actin protrusions. The number of cells showing GFP expression (infected cells, about 30%) was scored 24 h after addition of virus and the data was normalized to the unperturbed controls. The graph shows the relative amount of infected cells normalized to virus infection in the absence of blebbistatin. Error bars represent the standard deviation of three independent experiments. Numbers below the graph indicate unnormalized infection data. (A) HaCaT cells were transfected with EGFP-actin and subsequently inoculated with AF488 labeled HPV-16 PsV (100 particles/cell). 30 min after virus addition cells were imaged at 1 fps with spinning disc confocal microscopy. Kymograph shows pictures of a single actin protrusion of HaCaT cells from time series (5 s intervals) oriented such that tip of protrusions are at the top and the cell body at the bottom. (B) HeLa or HaCaT cells were infected with HPV-16 PsV expressing GFP (50 or 30 particles/cell, respectively) with or without inhibition of actin retrograde flow by blebbistatin. Cells were grown as confluent monolayers without the presence of long actin protrusions or subconfluently exhibiting frequently actin protrusions. The percentage of cells showing GFP expression (infected cells, 30–40%) was scored 36 h after addition of virus and the data was normalized to the unperturbed controls. The graph shows the relative amount of infected cells normalized to virus infection in the absence of blebbistatin. Error bars represent the standard deviation of three independent experiments. Numbers below the graph indicate unnormalized infection data (infection).

When actin retrograde flow was inhibited, virus particles could not be actively transported along actin rich protrusions. However, in confluent tissue culture and in epidermal tissues *in vivo* cells do not usually form long actin protrusions. Accordingly, HPV-16 infection of confluent HeLa cells was insensitive to inhibition of the actin retrograde flow-mediated transport by blebbistatin (Fig. 6B, white bars). This result suggested, in addition, that neither actin transport nor myosin II were required once viruses had bound directly to the cell body in contrast to other viruses such as vaccinia virus [38]. However, when subconfluent cells, that formed actin protrusions, were infected with HPV-16, and virus transport was blocked, infectivity was reduced by 36% (Fig. 6B, black bars).

HeLa cells were used throughout this study for ease of live cell microscopy. To address whether the observed phenomena also occurred in keratinocytes, we analyzed transport of viral particles along actin rich protrusions in HaCaT cells using the methods described previously. Directed transport and diffusion of HPV-16 PsV was observed on stable actin-rich protrusions. PsV exhibiting directed transport moved with a speed of 2±1 µm/min (Fig. 6A, n = 32; Video S16). Infection of confluent HaCaT cells was insensitive to blebbistatin inhibition, but infection of sparse HaCaT cells was reduced by 50% similar to what we observed in HeLa cells (Fig. 6B).

That infection was not entirely blocked by abrogation of transport was most likely due to a significant portion of virus particles binding directly to the cell body as opposed to actin rich protrusions. Particles binding directly to the cell body would be able to access the endocytic machinery for entry whereas particles binding far from the cell body would not. Hence, active transport was not required for infection but had the ability to facilitate infection, and increased the number of infected cells as compared to cells that had no transport (cells in the presence of blebbistatin). This may be an important factor for HPV infection in vivo, where the initial infection occurs only in basal keratinocytes most likely after wounding of the epidermal tissue, and where only few viruses may have access to the target cells.

## Discussion

The cell surface dynamics of HPV-16 indicated that the virus has co-opted a transport mechanism along actin rich cell protrusions to access the endocytic machinery present at the cell body, and thus to enhance infectious entry. The transport was facilitated by binding to receptors that, in turn, were likely to interact with actin filaments to mediate the transport towards the cell body powered by retrograde flow. This mechanism is not without precedent; several enveloped RNA viruses have been found to use a similar mechanism [3]. However, HPV-16 is the first nonenveloped virus found to use such a mechanism. It is to be expected that many other viruses are capable of using this cellular mechanism.

We found that certain dynamic properties of HPV-16 on the cell body are similar to murine Polyomavirus (mPy) particles: while some particles displayed random, diffusive motion most particles displayed rapid confinement [2]. It is interesting to note, that disruption of actin filaments eliminates the confinement of mPy, as if confinement would depend on a link with actin similar to what we observed for the transport of HPV-16 along actin protrusions. Alternatively, HPV-16 confinement on the cell body may be the result of binding simultaneously to both, extracelluar matrix components and cell surface receptors, or it may be due to limited virus receptor diffusion caused by actin dependent confinement zones [39]. While HPV-16 binds to diverse HSPGs [15]–[17], mPy binds the glycosphingolipids GD1a and GT1b [25],[26], which implies that these structurally distinct and differentially localized receptors exhibit similar diffusion properties. Hence, the diffusive properties of HPV-16 alone cannot be responsible for the unusually slow internalisation kinetics. However, we cannot exclude an interaction of HPV-16 with a putative secondary receptor that may be responsible for cell surface motion and/or subsequent internalization.

Our findings showed, however, that HPV-16 binding provided a cellular system where viruses were actively transported along actin protrusions towards the cell. This transport was specific to HPV-16, since SV40, a structurally similar nonenveloped DNA virus, displayed only random motion on the protrusions. HPV-16 transport was sensitive to inhibitors of actin polymerisation and depolymerisation, of myosin II and myosin light chain kinase, and of ATP synthesis. These properties are consistent with a transport mechanism based on actin retrograde flow [35],[36]. The study of Lehmann et al. [3] showed that murine leukemia virus shares the same properties. We could demonstrate, moreover, that HPV-16 transport coincided with the net transport of actin molecules in the respective cell protrusions, and that depending on the protrusion the movement of actin and the virus could each occur at two different rates.

All these findings supported a mechanism where viruses bind to a cellular receptor, which in turn must somehow bind to F-actin in order to be pulled by actin retrograde flow towards the cell body. Lehmann et al [3] showed that for transport of murine leukemia virus its receptor (mCAT-1) is clustered by the virus. Clustering of the receptor most likely combined with signal transduction elicited by this event would then provide the cue for a link to actin as has been proposed for the movement of anti-apCAM-antibody-coated beads. These beads cluster the cell adhesion molecule apCAM and are transported along filopodial growth cones by actin retrograde flow [40]. However, extensive clustering itself may not be a prerequisit for the signal transduction event. Using single molecule tracking of epidermal growth factor (EGF), Lidke et al. [31] showed that dimerization of receptors by an EGF molecule is sufficient to trigger transport along filopodia. That such ‚cues' existed for the transport of HPV-16, and that events leading to a link between the HPV-16-receptor complex and actin occurred, was suggested by the observed switch from diffusion to directed motion. However, switches were observed infrequently and asynchronously after binding of viruses to protrusions. Some particles displayed random motion for as long as 2 h after addition of HPV-16 PsV to cells, indicating that not all particles located on actin protrusions may encounter the ‚cue' for active transport.

The interaction of a receptor with actin may occur either directly by the receptor through its cytosolic tail or indirectly through binding to an actin binding protein. The receptor and its ligand–in this case HPV-16–would then be linked to actin retrograde flow. A variety of HSPGs can serve as binding receptors for infectious HPV-16 entry [17]. Of these, the syndecan family member are likely candidates as receptors for transport, as they in contrast to glypicans have a large cytosolic domain known to interact with actin binding proteins [41]–[43]. However, it cannot be excluded that other molecules in the plasma membrane serve as co-receptor for HPV-16, and that engagement of co-receptors provides the ‘cue’ for active transport.

Although our results favour a transport mechanism based on actin retrograde flow rather than transport by an unconventional myosin, some unanswered questions remain. Actin retrograde flow is the result of balanced actin polymerisation, depolymerisation, and myosin II function [29],[35]. Why did pertubation of one of the three processes abrogate directed virus transport altogether? When actin polymerisation is blocked, several studies show that cell extensions shrink due to continued myosin II function, actin depolymerisation, and actin retrograde flow. Actin depolymerisation agents and myosin II inhibitors, as expected, induce increased growth of actin protrusions. However, in all cases, retrograde flow is reduced, but not blocked [29],[35]. Thus, one would expect that viruses would be transported to the cell body, but at reduced speeds. We hypothesize that a reduction of force generation due to reduced actin retrograde flow weakens the connection between actin and the receptor, until the connection is lost and viruses start to diffuse again. Our hypothesis is supported by the finding that when weak forces as low as 10–50 pN are applied to receptor-actin linkages such as the fibronectin-integrin-actin link or the cell adhesion molecule apCAM-actin link the interaction is strengthened [40],[44].

A variety of cell surface molecules such as members of the immunoglobulin family (apCAM), the EGF receptor, mCAT-1, the receptor that facilitates murine leukemia virus entry, or potentially HSPGs support transport of ligands along filopodia [3],[30],[31],[40]. The mobility of filopodia and the retrograde transport of receptors allow the cell to sense its environment. In sparse tissue culture, viruses can use receptor transport along filopodia, gain access to cellular entry sites, and thus enhance the probability of infection. In dense cultures, our results show that the effect is not important. During in vivo transmission of HPV-16, infectious particles are shed after abrasion of terminally differentiated, infected keratinocytes. Viruses will be able to access the target cells (basal keratinocytes) most likely in wounded tissue [5]. Since wounding of mucosal or skin epidermis results in upregulation of syndecan-1, a HPV-16 receptor candidate [17],[45],[46], and in filopodia formation of the basal keratinocytes that it is essential for reepitheliasation [47], HPV-16 could use actin retrograde flow for efficient transport towards entry sites on the cell body and thus facilitate infection.

Clearly, many interesting questions remain: How does actin retrograde transport of HPV-16 translate into kinetics of infection? Are the viruses internalized immediately after reaching the cell body? How important is actin retrograde transport in vivo? How many other viruses use this translocation system? Is there a common linker protein between viral receptors and actin?

## Materials and Methods

### Cells, viruses, and drugs

HeLa and HaCaT cells were cultured in DMEM (Invitrogen) containing 10% fetal calf serum. (-)-Blebbistatin, cytochalasin D, ML-7, nocodazole, and sodium azide were from Sigma. Jasplakinolide was from Molecular Probes. HPV-16 PsV containing the pCIneo-mRFP plasmid was produced with the p16L1L2 plasmid by the propagation method described by Buck and Thompson [48]. The PsV was matured for 24 hours in the presence of RNase A to maximize the purification of pseudovirions containing the reporter plasmid, resulting in an improved particle to infectivity ratio [48]. All plasmids and production methods are fully described on the Schiller laboratory's website (http://ccr.cancer.gov/staff/staff.asp?profileid=5637). SV40 was produced as described [49].

### Plasmids

The plasmid pEGFP-actin was from Clontech. The plasmid pmPAGFP-actin was constructed as follows: the EGFP sequence was excised from pEGFP-actin by NheI/XhoI digestion and replaced by ligation of the mPAGFP sequence from pmPAGFP (kind gift of George H. Patterson, NIH, Bethesda, USA).

### Fluorescent labeling of HPV-16 PsV and of SV40

SV40 was covalently labeled with fluorophores as described [49]. HPV-16 PsV labeling followed essentially the same protocol. Briefly, purified HPV-16 PsV were incubated for 1 h at room temperature in PBS with a ten-fold molar excess of Fluorescein or Alexa Fluor (AF) succinimidylesters (Molecular Probes) over the major capsid protein L1. PsV were separated from the labeling reagent by size exclusion chromatography using NAP5 columns (GE Healthcare) and stored at 4°C. The degree of labeling (DOL) was determined by spectophotometry using DOL = (A_max_×MW)/([protein]×e_dye_), with A_max_ = absorbance of dye at absorbance maximum, MW = molecular weight of a virus particle, [protein] = protein concentration, and e_dye_ = extinction coefficient of the dye at its absorbance maximum. Please refer to the manufacturer's instructions for further details.

### Transient expression

For transfection, cells were trypsinized, pelleted, washed with PBS and transfected with expression plasmids in Nucleofector solution R (Amaxa) utilizing program I13 of the Amaxa Nucleofector according to the manufacturer's instructions. Cells were seeded on 18 mm coverslips and used for live cell imaging experiments at 6–14 h post transfection.

### Single particle tracking

Microscopy was performed on a custom modified Olympus IX71 inverted microscope. Modifications included a heated incubation chamber that surrounded the microscope stage set to 37°C, an objective-type total internal reflection fluorescence microscopy setup from TILL Photonics (Grafeling, Germany), and a monochromator for epifluorescence excitation with a controller allowing hardware-controlled fast switching between total internal reflection fluorescence and epi-fluorescence excitation and acquisition (TILL Photonics). Images were acquired using a TILL Image QE chargecoupled device camera and TILLVISION software (both from TILL Photonics). The total internal reflection angle was manually adjusted for every experiment. Live HeLa cells on 18-mm coverslips were mounted in custom-made chambers. To avoid changes in membrane or cytoskeleton, the medium was not exchanged when mounting cells. HPV-16 PsV were added at 0.1 mg/ml into the 0.5 ml of medium on the stage. Movies were recorded at a rate of 20 frames per s for 1,000 or 2,000 frames in TIRF mode. After each experiment, the cells that had been recorded were imaged by differential interference contrast microscopy to check for viability. Trajectories were harvested and analyzed using a tracking program [24]. The position accuracy for the particles allowed by this program was on average 26 nm (see Figure S1).

### Live cell imaging of virus transport and image processing

Epifluorescence microscopy was performed on a Zeiss 200 M inverted microscope with a heated objective and a heated stage holding the cell chamber. Cells and objective were kept at 37°C, and cells were incubated in CO2-independent medium (Invitrogen). Images were acquired with Openlab Software (Improvision).

Spinning disc confocal microscopy was performed on a Zeiss 200 M microscope with a Visitech spinning disc setup. The spinning disc confocal microscope was equipped with heated incubation chamber that surrounded the microscope stage set at 37°C. Images were acquired with MetaMorph Software (Visitron).

Live cells that had been transfected with EGFP-actin were mounted on 18-mm coverslips in custom-made chambers, and cells were incubated with normal growth medium. HPV-16 PsV were added at 0.1 mg/ml into the 0.5 ml of medium on the stage. After 5 min–2 h of binding virus particles to cells image acquisition was performed. Inhibitors were always added directly into the medium during acuisition of images to the final concentrations given. Images were imported into ImageJ (NIH) and kymographs of single actin protrusions were assembled showing every second or fourth image as indicated in the figure legends.

### Imaging of actin retrograde flow

Microscopy was performed on a Leica SP2 AOBS scanning confocal microscope equipped with a solid state laser (405 nm excitation) and a heated incubation chamber that surrounded the microscope stage set at 37°C. Live HeLa cells that been transfected with pmPAGFP-actin on 18-mm coverslips were mounted in custom-made chambers, and cells were incubated with normal growth medium. After a 500 ms pulse of 405 nm light (either point or frame), images were acquired at 0.5 Hz.

### Electron microscopy

HPV-16 PsV (300 ng) were added to 1×10^5^ cells for 10 min or 1 h at 37°C, and unbound virus was removed prior to fixation with 2% glutaraldehyde / 2% osmium tetroxide. Sample preparation and thin section electron microscopy was performed according to standard electron microscopy procedures. For negative staining, 0.4 mm mesh copper grids were coated with a 4 nm carbon film. Sample containing AF488 labeled or unlabeled HPV-16 PsV was added for 30 s, drained of excess liquid, and stained for an additional 30 s with 2% uranyl acetate in distilled water. After transmission electron microscopy, images were exported as 8-bit TIFF files and processed in PHOTOSHOP 8.0 (Adobe Systems).

### Infection studies

HeLa cells were seeded on 24 h prior to experimentation to result in 20 or 100 % confluency. HPV-16 PsV were added to cells with or without pretreatment of blebbistatin (30 min.) at 0.1 transducing particles/cell (20 ng or 100 ng) to result in 30%–40% XFP expressing cells in the unperturbed control. 24 h after addition of virus, cells were trypsinized, fixed in 4% formaldehyde, and analyzed for XFP expression by flow cytometric analysis.

## Supporting Information

Figure S1 The mobility of HPV 16 particles immobilized on coverglass. The position accuracy of the tracking software is dependent upon the signal to noise ratio for the individual particle. The signal to noise ratio is different for each particle in each frame and a theoretical position accuracy can be calculated for each particle and frame. The performance of the particle tracking software is described in detail in [2],[24]. Since other factors such as vibrations of the microscope stage contribute to the position accuracy of the actual measurement, we performed single particle tracking of HPV particles attached to coverglass that considered them immobile. Particles with a mobility comparable to that of these particles were discarded from analysis. The radius of the area covered by trajectories of particles adsorbed to the coverglass was taken as position accuracy. Shown is a scatter plot of the diffusion coefficient versus the slope of the moment scaling spectrum (S_MSS_) of HPV-16 PsV trajectories on coverglass. Every point represents one trajectory. The black circles represent individual particles bound to coverglass imaged and analyzed in the same way as particles bound to cells. The red error bars represent the standard deviation of the mobility of such particles. The particles at the left edge exhibit a negative D, so that the imaging noise completely obscures the observable mobility.
(5.2 MB TIF)

Video S1 Directed movement of HPV-16 on the cell body. Movie from a TIRF-M time series showing one AF488 labeled HPV-16 particle in directed, ballistic motion with the assigned trajectory on a HeLa cell over 288 frames played at realtime (14.4 s). Particle corresponds to Fig. 2C, D, 1.
(2.5 MB MOV)

Video S2 Diffusive movement of HPV-16 on the cell body. Movie from a TIRF-M time series showing one AF488 labeled HPV-16 particle in diffusive motion with the assigned trajectory on a HeLa cell over 354 frames played at realtime (17.7 s). Particle corresponds to Fig. 2C, D, 2.
(3.7 MB MOV)

Video S3 Slow drift of HPV-16 on the cell body. Movie from a TIRF-M time series showing one AF488 labeled HPV-16 particle with a slow drift with the assigned trajectory on a HeLa cell over 969 frames played at realtime (48.45 s). Particle corresponds to Fig. 2C, D, 3.
(3.3 MB MOV)

Video S4 Confined HPV-16 particle on the cell body. Movie from a TIRF-M time series showing one confined AF488 labeled HPV-16 particle with the assigned trajectory on a HeLa cell over 1561 frames played at realtime (78.05 s). Particle corresponds to Fig. 2C, D, 4.
(6.4 MB MOV)

Video S5 HPV-16 particles on the cell body. Overview movie from a TIRF-M time series showing several AF488 labeled HPV-16 particles on a HeLa cell over 2000 frames played at realtime (100 s). Particles correspond to Fig. 2B, C.
(5.0 MB MOV)

Video S6 HPV-16 motion on actin-rich protrusions of HeLa cells. Movie from an epifluorescence microscopy time series showing several AF488 labeled HPV-16 particles moving along actin-rich protrusions on HeLa cells expressing EGFP-actin. Acquisition of images occured at 2 frames per s, movie played at 10 Hz.
(8.2 MB MOV)

Video S7 HPV-16 moves along actin protrusions extracellularly. Movie from an epifluorescence microscopy time series showing several FITC labeled HPV-16 particles moving along protrusions on HeLa cells. 100 s after image acqusition start the extracellular medium was acidified to pH 5.5 as indicated by the white flash. Note, that the fluorescence of all particles was quenched indicating their extracellular localization. Acquisition of images occured at 2 frames per s, movie played at 20 Hz.
(2.3 MB MOV)

Video S8 SV40 diffusive motion on actin rich protrusions. Movie from a spinning disc confocal microscopy time series showing one AF488 labeled SV40 particle moving along an actin-rich protrusion on HeLa cells expressing EGFP-actin. Note, that the particle detached from the protrusion towards the end of the movie indicating the relatively weak affinity for binding. Acquisition of images occured at 5 frames per s, movie played at 10 Hz.
(1.2 MB MOV)

Video S9 Actin retrograde flow. Movie from laser scanning confocal microscopy time series showing several activated PAGFP spots in protrusions of HeLa cells transfected with PAGFP-actin after spot activation by 405 nm wavelength light. Note, that all spots move towards the cell body. Acquisition of images occured at 1 frames per 2 s, movie played at 5 Hz.
(0.49 MB MOV)

Video S10 Effect of nocodazole on virus transport. Movie from spinning disc confocal microscopy time series showing several AF488 labeled HPV-16 particles on an actin-rich protrusion of HeLa cells transfected with EGFP-actin. Addition of nocodazole to 5 µM concentration is indicated by a white flash. Acquisition of images occured at 4 frames per s, movie played at 10 Hz.
(0.90 MB MOV)

Video S11 Effect of cytochalasin D on virus transport. Movie from spinning disc confocal microscopy time series showing several AF488 labeled HPV-16 particles on an actin-rich protrusion of HeLa cells transfected with EGFP-actin. Addition of cytochalasin D to 2 µM concentration is indicated by a white flash. Acqusition of images occured at 4 frames per s, movie played at 10 Hz.
(0.64 MB MOV)

Video S12 Effect of cytochalasin D on virus transport (overview). Movie from spinning disc confocal microscopy time series showing several AF488 labeled HPV-16 particles on actin-rich protrusions of a HeLa cell transfected with EGFP-actin. Addition of cytochalasin D to 2 µM concentration is indicated by a white flash. Acquisition of images occured at 4 frames per s, movie played at 10 Hz.
(13.6 MB MOV)

Video S13 Effect of jasplakinolide on virus transport. Movie from spinning disc confocal microscopy time series showing several AF488 labeled HPV-16 particles on an actin-rich protrusion of HeLa cells transfected with EGFP-actin. Addition of jasplakinolide to 300 nM concentration is indicated by a white flash. Acquisition of images occured at 4 frames per s, movie played at 10 Hz.
(0.65 MB MOV)

Video S14 Effect of blebbistatin on virus transport. Movie from spinning disc confocal microscopy time series showing several AF488 labeled HPV-16 particles on an actin-rich protrusion of HeLa cells transfected with EGFP-actin. Addition of blebbistatin to 30 µM concentration is indicated by a white flash. Acquisition of images occured at 4 frames per s, movie played at 10 Hz.
(0.34 MB MOV)

Video S15 Effect of blebbistatin on virus transport (overview). Movie from spinning disc confocal microscopy time series showing several AF488 labeled HPV-16 particles on actin-rich protrusions of a HeLa cell transfected with EGFP-actin. Addition of blebbistatin to 30 µM concentration is indicated by a white flash. Acquisition of images occured at 4 frames per s, movie played at 10 Hz.
(13.5 MB MOV)

Video S16 HPV-16 motion on actin-rich protrusions of HaCaT cells. Movie from an epifluorescence microscopy time series showing several AF488 labeled HPV-16 particles moving along actin-rich protrusions on HaCaT cell expressing EGFP-actin. Acqusition of images occured at 1 frames per s, movie played at 5 Hz.
(7.0 MB MOV)
