# Supplementary figures and images for: Human Papillomavirus Type 16 Entry: Retrograde Cell Surface Transport along Actin-Rich Protrusions
Source: PLoS Pathog. 2008 Sep 5;4(9):e1000148. doi: 10.1371/journal.ppat.1000148 (PMC2518865; doi:10.1371/journal.ppat.1000148)

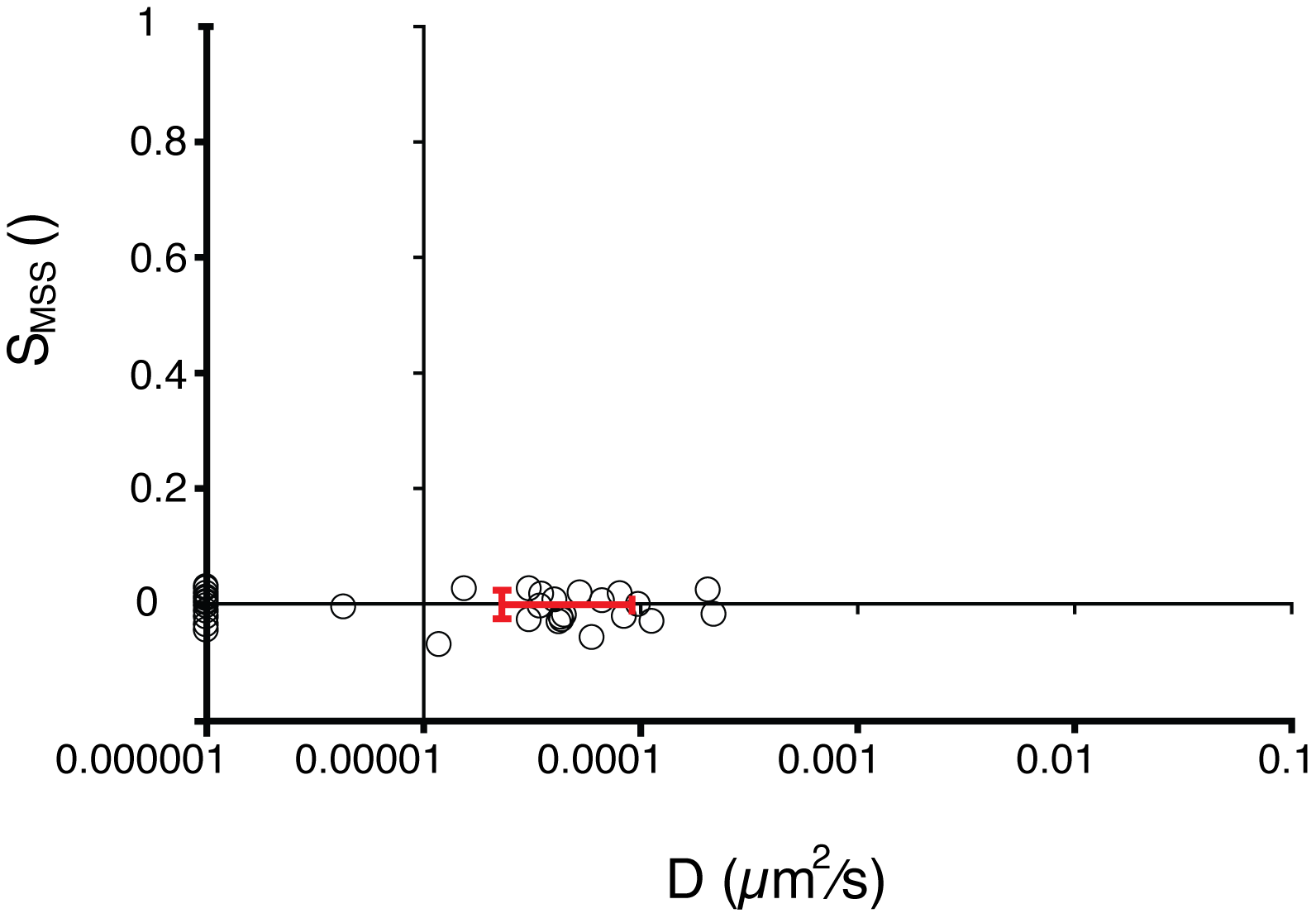

Supplement: Figure S1 — The mobility of HPV 16 particles immobilized on coverglass. The position accuracy of the tracking software is dependent upon the signal to noise ratio for the individual particle. The signal to noise ratio is different for each particle in each frame and a theoretical position accuracy can be calculated for each particle and frame. The performance of the particle tracking software is described in detail in [2],[24]. Since other factors such as vibrations of the microscope stage contribute to the position accuracy of the actual measurement, we performed single particle tracking of HPV particles attached to coverglass that considered them immobile. Particles with a mobility comparable to that of these particles were discarded from analysis. The radius of the area covered by trajectories of particles adsorbed to the coverglass was taken as position accuracy. Shown is a scatter plot of the diffusion coefficient versus the slope of the moment scaling spectrum (SMSS) of HPV-16 PsV trajectories on coverglass. Every point represents one trajectory. The black circles represent individual particles bound to coverglass imaged and analyzed in the same way as particles bound to cells. The red error bars represent the standard deviation of the mobility of such particles. The particles at the left edge exhibit a negative D, so that the imaging noise completely obscures the observable mobility. (5.2 MB TIF) [file ppat.1000148.s001.tif]
